# *catena*-Poly[[dichloridonickel(II)]-μ-1,3-di-4-pyridylpropane]

**DOI:** 10.1107/S1600536808018862

**Published:** 2008-06-28

**Authors:** Chun-Sheng Zhou, Guo-Chun Zhang

**Affiliations:** a Department of Chemistry, ShangLuo University, ShangLuo, Shaanxi 726000, People’s Republic of China

## Abstract

The title compound, [NiCl_2_(C_13_H_14_N_2_)]_*n*_, is a one-dimensional polymer built up from alternating NiCl_2_ units and bridging 1,3-di-4-pyridylpropane ligands. The Ni atom has a distorted tetra­hedral coordination formed by the Cl atoms and two N atoms from two ligands.  A mirror plane pases through the central methylene group of the propyl chain.

## Related literature

For a closely related structure, see: Zhang & Yu (2007 ▶). For related literature, see: Carlucci *et al.* (2002 ▶); Hennigar *et al.* (1997 ▶); Yaghi *et al.* (1998 ▶); Dalbavie *et al.* (2002 ▶); Ghosh *et al.* (2006 ▶); Marshall & Grushin (2005 ▶); Masood *et al.* (1994 ▶); McConnell & Nuttall (1978 ▶); Wu *et al.* (1999 ▶).
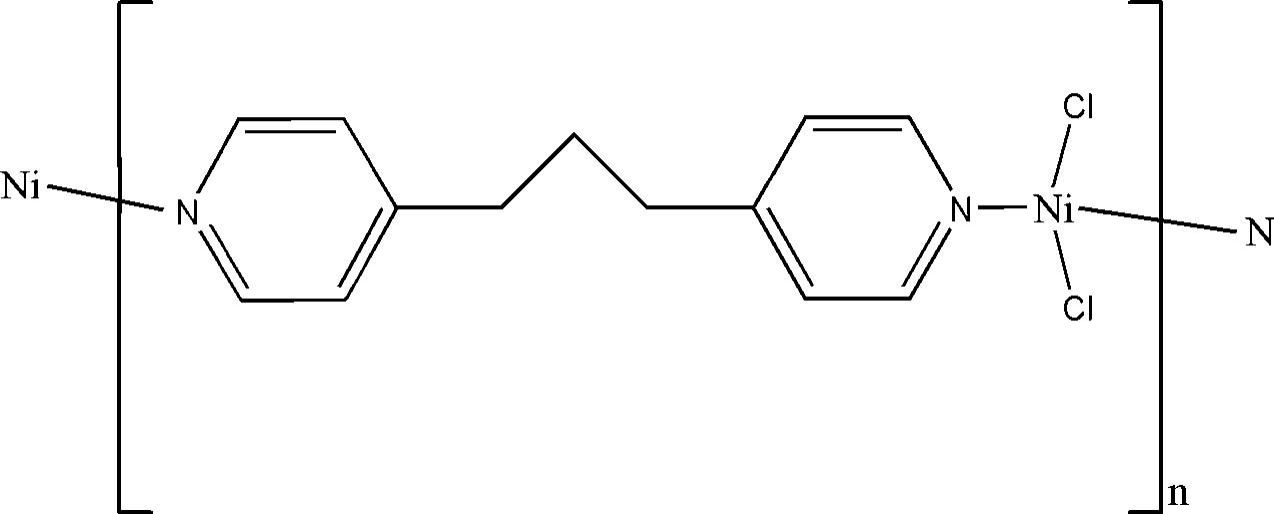


## Experimental

### 

#### Crystal data

[NiCl_2_(C_13_H_14_N_2_)]
*M* *_r_* = 327.87
Monoclinic, 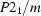

*a* = 5.1928 (17) Å
*b* = 12.972 (4) Å
*c* = 10.492 (3) Å
β = 93.588 (6)°
*V* = 705.3 (4) Å^3^
*Z* = 2
Mo *K*α radiation
μ = 1.74 mm^−1^
*T* = 298 (2) K
0.25 × 0.20 × 0.16 mm

#### Data collection

Bruker APEX area-detector diffractometer
Absorption correction: multi-scan (*SADABS*; Sheldrick, 1996 ▶) *T*_min_ = 0.671, *T*_max_ = 0.769
3581 measured reflections
1328 independent reflections
763 reflections with *I* > 2σ(*I*)
*R*_int_ = 0.046

#### Refinement

*R*[*F* ^2^ > 2σ(*F* ^2^)] = 0.038
*wR*(*F* ^2^) = 0.067
*S* = 0.87
1328 reflections
88 parameters
H-atom parameters constrained
Δρ_max_ = 0.44 e Å^−3^
Δρ_min_ = −0.44 e Å^−3^

### 

Data collection: *SMART* (Bruker, 1998 ▶); cell refinement: *SAINT* (Bruker, 1999 ▶); data reduction: *SAINT*; program(s) used to solve structure: *SHELXS97* (Sheldrick, 2008 ▶); program(s) used to refine structure: *SHELXL97* (Sheldrick, 2008 ▶); molecular graphics: *SHELXTL* (Sheldrick, 2008 ▶); software used to prepare material for publication: *SHELXTL*.

## Supplementary Material

Crystal structure: contains datablocks I, global. DOI: 10.1107/S1600536808018862/dn2360sup1.cif

Structure factors: contains datablocks I. DOI: 10.1107/S1600536808018862/dn2360Isup2.hkl

Additional supplementary materials:  crystallographic information; 3D view; checkCIF report

## Figures and Tables

**Fig. 1.**
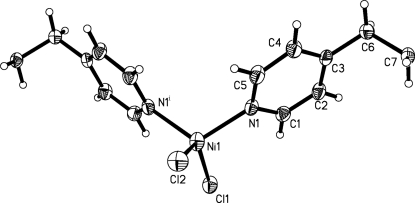
The structure of (I), showing the atom labellinging scheme. Displacement ellipsoids are drawn at the 30% probability level. H atoms are represented as small spheres of arbitrary radii.

## supplementary crystallographic information

### Comment

Recent years have seen the evolution of a new class of coordination polymers known collectively as metal organic framework materials (Yaghi *et al.*, 1998). The most common approach for producing coordination polymers and metal organic framework materials is through the self-assembly of metal centers with appropriate organic linker species to promote extended topologies (Hennigar *et al.*, 1997). Conformationally flexibly ligands are typical building elements in the molecular interlocked/intertwined species. Some work on the self-assembly of coordination networks have been reported in the presence of 1,3-di-4-pyridylpropane (bpp) ligand (Carlucci *et al.*, 2002). In this paper, we report here the synthesis and crystal structure of the title compound (I).

The Ni atom in the title complex has a distorted tetrahedral coordination formed by the chlorine atoms and two nitrogen from two separate bpp ligands (Fig. 1). The distances of Ni1—Cl and Ni1—Cl are 2.2533 (17) and 2.2382 (16)Å , respectively. Figure 1 show that this one-dimensional polymer built up from alternating (NiCl_2_) units and bridging 1,3-di-4-pyridylpropane ligands. Some other NiCl_2_ complexes with tetrahedral coordination geometries have been reported (Wu *et al.*, 1999; Dalbavie *et al.*, 2002; Masood *et al.*, 1994; McConnell & Nuttall, 1978 ; Ghosh *et al.*, 2006; Marshall & Grushin, 2005).

### Experimental

Bpp (0.21, 0.1 mmol), NiCl_2_ (0.22 g, 0.012 mmol), were added in a mixed solvent of methanol and acetonitrile, the mixture was heated for six hours under reflux. during the process stirring and influx were required. The resultant was then filtered to give a pure solution which was infiltrated by diethyl ether freely in a closed vessel, a weeks later some single crystals of the size suitable for X-Ray diffraction analysis.

### Refinement

All H atoms were fixed geometrically and treated as riding with C—H = 0.93 Å (aromatic) and U_iso_(H) = 1.2U_eq_(C).

### Crystal data

**Table:**

[NiCl_2_(C_13_H_14_N_2_)]	*F*_000_ = 336
*M**_r_* = 327.87	*D*_x_ = 1.544 Mg m^−^^3^
Monoclinic, *P*2_1_/*m*	Mo *K*α radiation λ = 0.71073 Å
Hall symbol: -P 2yb	Cell parameters from 1328 reflections
*a* = 5.1928 (17) Å	θ = 2.5–25.2º
*b* = 12.972 (4) Å	µ = 1.74 mm^−^^1^
*c* = 10.492 (3) Å	*T* = 298 (2) K
β = 93.588 (6)º	Bloc, green
*V* = 705.3 (4) Å^3^	0.25 × 0.20 × 0.16 mm
*Z* = 2	

### Data collection

**Table:**

Bruker APEX area-detector diffractometer	1328 independent reflections
Radiation source: fine-focus sealed tube	763 reflections with *I* > 2σ(*I*)
Monochromator: graphite	*R*_int_ = 0.046
*T* = 298(2) K	θ_max_ = 25.2º
φ and ω scans	θ_min_ = 2.5º
Absorption correction: multi-scan(SADABS; Sheldrick, 1996)	*h* = −6→5
*T*_min_ = 0.671, *T*_max_ = 0.769	*k* = −13→15
3581 measured reflections	*l* = −12→12

### Refinement

**Table:**

Refinement on *F*^2^	Secondary atom site location: difference Fourier map
Least-squares matrix: full	Hydrogen site location: inferred from neighbouring sites
*R*[*F*^2^ > 2σ(*F*^2^)] = 0.038	H-atom parameters constrained
*wR*(*F*^2^) = 0.067	*w* = 1/[σ^2^(*F*_o_^2^) + (0.009*P*)^2^ + 0.821*P*] where *P* = (*F*_o_^2^ + 2*F*_c_^2^)/3
*S* = 0.87	(Δ/σ)_max_ < 0.001
1328 reflections	Δρ_max_ = 0.44 e Å^−^^3^
88 parameters	Δρ_min_ = −0.44 e Å^−^^3^
Primary atom site location: structure-invariant direct methods	Extinction correction: none

### Special details

**Table:**

Geometry. All e.s.d.'s (except the e.s.d. in the dihedral angle between two l.s. planes) are estimated using the full covariance matrix. The cell e.s.d.'s are taken into account individually in the estimation of e.s.d.'s in distances, angles and torsion angles; correlations between e.s.d.'s in cell parameters are only used when they are defined by crystal symmetry. An approximate (isotropic) treatment of cell e.s.d.'s is used for estimating e.s.d.'s involving l.s. planes.
Refinement. Refinement of *F*^2^ against ALL reflections. The weighted *R*-factor *wR* and goodness of fit *S* are based on *F*^2^, conventional *R*-factors *R* are based on *F*, with *F* set to zero for negative *F*^2^. The threshold expression of *F*^2^ > σ(*F*^2^) is used only for calculating *R*-factors(gt) *etc*. and is not relevant to the choice of reflections for refinement. *R*-factors based on *F*^2^ are statistically about twice as large as those based on *F*, and *R*- factors based on ALL data will be even larger.

### Fractional atomic coordinates and isotropic or equivalent isotropic displacement parameters (Å^2^)

**Table:**

	*x*	*y*	*z*	*U*_iso_*/*U*_eq_	
Ni1	0.45214 (16)	0.7500	0.22234 (6)	0.0554 (3)	
Cl1	0.7422 (3)	0.7500	0.39091 (12)	0.0566 (4)	
Cl2	0.5444 (3)	0.7500	0.01676 (12)	0.0654 (5)	
N1	0.2418 (6)	0.6191 (2)	0.2414 (3)	0.0443 (8)	
C1	0.2869 (7)	0.5560 (3)	0.3416 (3)	0.0501 (10)	
H1	0.4209	0.5719	0.4013	0.060*	
C3	−0.0579 (7)	0.4430 (3)	0.2738 (3)	0.0445 (10)	
C5	0.0501 (8)	0.5930 (3)	0.1581 (3)	0.0555 (11)	
H5	0.0171	0.6349	0.0871	0.067*	
C4	−0.1015 (8)	0.5079 (3)	0.1707 (3)	0.0546 (11)	
H4	−0.2344	0.4939	0.1097	0.066*	
C2	0.1436 (8)	0.4690 (3)	0.3594 (3)	0.0521 (11)	
H2	0.1827	0.4271	0.4300	0.063*	
C7	−0.0598 (10)	0.2500	0.2643 (4)	0.0462 (14)	
H7A	−0.0175	0.2500	0.1755	0.055*	
H7B	0.1005	0.2500	0.3169	0.055*	
C6	−0.2131 (7)	0.3473 (3)	0.2915 (3)	0.0521 (11)	
H6A	−0.3677	0.3495	0.2347	0.063*	
H6B	−0.2658	0.3448	0.3786	0.063*	

### Atomic displacement parameters (Å^2^)

**Table:**

	*U*^11^	*U*^22^	*U*^33^	*U*^12^	*U*^13^	*U*^23^
Ni1	0.0726 (7)	0.0426 (4)	0.0503 (4)	0.000	−0.0005 (4)	0.000
Cl1	0.0671 (12)	0.0479 (8)	0.0534 (8)	0.000	−0.0083 (7)	0.000
Cl2	0.0733 (13)	0.0780 (10)	0.0444 (8)	0.000	−0.0006 (7)	0.000
N1	0.052 (2)	0.0332 (17)	0.0466 (17)	0.0034 (16)	−0.0023 (16)	−0.0024 (14)
C1	0.050 (3)	0.047 (2)	0.052 (2)	0.003 (2)	−0.0080 (19)	0.0003 (19)
C3	0.049 (3)	0.030 (2)	0.055 (2)	0.006 (2)	0.003 (2)	−0.0070 (17)
C5	0.070 (3)	0.042 (2)	0.053 (2)	0.003 (2)	−0.009 (2)	0.0027 (19)
C4	0.062 (3)	0.047 (2)	0.052 (2)	0.001 (2)	−0.014 (2)	−0.0052 (19)
C2	0.066 (3)	0.039 (2)	0.050 (2)	0.005 (2)	−0.007 (2)	0.0072 (18)
C7	0.054 (4)	0.034 (3)	0.051 (3)	0.000	0.000 (3)	0.000
C6	0.046 (3)	0.043 (2)	0.068 (3)	−0.001 (2)	0.004 (2)	−0.0085 (19)

### Geometric parameters (Å, °)

**Table:**

Ni1—N1	2.036 (3)	C5—C4	1.366 (5)
Ni1—N1^i^	2.036 (3)	C5—H5	0.9300
Ni1—Cl2	2.2384 (16)	C4—H4	0.9300
Ni1—Cl1	2.2503 (16)	C2—H2	0.9300
N1—C5	1.327 (4)	C7—C6^ii^	1.529 (4)
N1—C1	1.341 (4)	C7—C6	1.529 (4)
C1—C2	1.372 (5)	C7—H7A	0.9700
C1—H1	0.9300	C7—H7B	0.9700
C3—C2	1.377 (5)	C6—H6A	0.9700
C3—C4	1.378 (4)	C6—H6B	0.9700
C3—C6	1.499 (5)		
			
N1—Ni1—N1^i^	113.06 (17)	C5—C4—C3	120.1 (4)
N1—Ni1—Cl2	104.07 (8)	C5—C4—H4	119.9
N1^i^—Ni1—Cl2	104.07 (8)	C3—C4—H4	119.9
N1—Ni1—Cl1	105.05 (9)	C1—C2—C3	120.5 (3)
N1^i^—Ni1—Cl1	105.05 (9)	C1—C2—H2	119.7
Cl2—Ni1—Cl1	125.74 (7)	C3—C2—H2	119.7
C5—N1—C1	116.6 (3)	C6^ii^—C7—C6	111.3 (4)
C5—N1—Ni1	122.2 (2)	C6^ii^—C7—H7A	109.4
C1—N1—Ni1	121.1 (3)	C6—C7—H7A	109.4
N1—C1—C2	122.6 (3)	C6^ii^—C7—H7B	109.4
N1—C1—H1	118.7	C6—C7—H7B	109.4
C2—C1—H1	118.7	H7A—C7—H7B	108.0
C2—C3—C4	116.3 (4)	C3—C6—C7	111.7 (3)
C2—C3—C6	120.9 (3)	C3—C6—H6A	109.3
C4—C3—C6	122.7 (4)	C7—C6—H6A	109.3
N1—C5—C4	123.7 (3)	C3—C6—H6B	109.3
N1—C5—H5	118.1	C7—C6—H6B	109.3
C4—C5—H5	118.1	H6A—C6—H6B	107.9

Symmetry codes: (i) *x*, −*y*+3/2, *z*; (ii) *x*, −*y*+1/2, *z*.
